# Effect of Hydrophobic Mutations in the H2-H3 Subdomain of Prion Protein on Stability and Conversion *In Vitro* and *In Vivo*

**DOI:** 10.1371/journal.pone.0024238

**Published:** 2011-09-01

**Authors:** Iva Hafner-Bratkovič, Lars Gaedtke, Andrej Ondracka, Peter Veranič, Ina Vorberg, Roman Jerala

**Affiliations:** 1 Department of Biotechnology, National Institute of Chemistry, Ljubljana, Slovenia; 2 Institute of Virology, Technical University Munich, München, Germany; 3 Institute of Cell Biology, School of Medicine, University of Ljubljana, Ljubljana, Slovenia; 4 German Centre for Neurodegenerative Diseases (DZNE), Bonn, Germany; 5 Faculty of Chemistry and Chemical Technology, University of Ljubljana, Ljubljana, Slovenia; 6 EN→FIST Centre of Excellence, Ljubljana, Slovenia; Creighton University, United States of America

## Abstract

Prion diseases are fatal neurodegenerative diseases, which can be acquired, sporadic or genetic, the latter being linked to mutations in the gene encoding prion protein. We have recently described the importance of subdomain separation in the conversion of prion protein (PrP). The goal of the present study was to investigate the effect of increasing the hydrophobic interactions within the H2-H3 subdomain on PrP conversion. Three hydrophobic mutations were introduced into PrP. The mutation V209I associated with human prion disease did not alter protein stability or *in vitro* fibrillization propensity of PrP. The designed mutations V175I and T187I on the other hand increased protein thermal stability. V175I mutant fibrillized faster than wild-type PrP. Conversion delay of T187I was slightly longer, but fluorescence intensity of amyloid specific dye thioflavin T was significantly higher. Surprisingly, cells expressing V209I variant exhibited inefficient proteinase K resistant PrP formation upon infection with 22L strain, which is in contrast to cell lines expressing wild-type, V175I and T187I mPrPs. In agreement with increased ThT fluorescence at the plateau T187I expressing cell lines accumulated an increased amount of the proteinase K-resistant prion protein. We showed that T187I induces formation of thin fibrils, which are absent from other samples. We propose that larger solvent accessibility of I187 in comparison to wild-type and other mutants may interfere with lateral annealing of filaments and may be the underlying reason for increased conversion efficiency.

## Introduction

Prion diseases are neurodegenerative diseases characterized by misfolding of prion protein leading to pathologic amyloid deposits in brains of humans and other mammals. Cellular prion protein is composed of an N-terminal unstructured part and a globular C-terminal domain, composed of three α-helices (H1, H2, H3) and an antiparallel two-stranded β-sheet (B1, B2). This protein fold, which is conserved in all vertebrate prion proteins with determined structure, is stabilized by a tightly packed hydrophobic core, salt bridges and hydrogen bonds [1]. During the course of the disease, cellular prion protein (PrPC) undergoes conformational conversion into its pathological aggregated form, PrPSc. In humans, prion diseases can be sporadic, acquired or genetic, linked to mutations in the gene encoding prion protein, *PRNP*. In the globular domain over 20 different mutations in *PRNP* have been associated with familial forms of prion disease, familial Creutzfeldt-Jakob disease (fCJD), Gerstmann-Sträussler-Scheinker syndrome (GSS), and fatal familial insomnia (FFI) [2]. Some of these mutations are in the hydrophobic core and about one third of the mutations are substitutions for an amino acid with increased hydrophobicity. Several mechanisms have been proposed to explain how certain point mutations might modulate protein misfolding, such as decreased thermodynamic stability of PrP^C^ [3], [4], increased stability of the folding intermediate (for V210I) [5] or differences in posttranslational modifications and cellular trafficking such as atypical glycosylation of V180I and T183A [6], [7]. The mechanism of PrP^C^ to PrP^Sc^ structural conversion is to a large extent still unknown; particularly since the high resolution structure of PrP^Sc^ could not be resolved. Nevertheless, based on biochemical and biophysical characterization of PrP^Sc^ aggregates and *in vitro* prepared PrP fibrils, several structural models of PrP^Sc^ were proposed [8], [9], [10], [11]. We recently demonstrated, using disulfide tethering, that the hydrophobic core of the structured C-terminal domain is affected since the subdomains B1-H1-B2 and H2-H3 must separate during PrP conversion [11], [12]. However, disulfide tethers within subdomains did not prevent conversion, suggesting domain swapping as the process underlying PrP conversion [11].

The aim of the present study was to investigate the effect of the enhancement of the hydrophobic core of the H2-H3 globular subdomain on the PrP fibrillization capacity and its conversion into proteinase K resistant PrP (PrPres). Appropriately selected hydrophobic mutants could increase the stability of the globular domain or its subdomains and we wanted to investigate if increasing the hydrophobicity had any effect on the fibrillization propensities of these types of PrP mutants. Furthermore, cell culture experiment was performed to reveal if PrP mutants could form PrPres and support infection. Three hydrophobic mPrP (mouse prion protein) mutants were selected, prepared and correctly refolded. One of these mutants, T187I, demonstrated increased formation of thin PrP fibrils *in vitro* and in cell culture increased proteinase K-resistant PrP formation upon 22L prion strain infection despite its increased stability. This mutant might prove as a valuable substrate for *in vitro* seeding assays [13] and scrapie cell assays [14].

## Results

### Selection of sites for introduction of hydrophobic residues and their influence on thermal stability of mPrP

**Figure 1:**
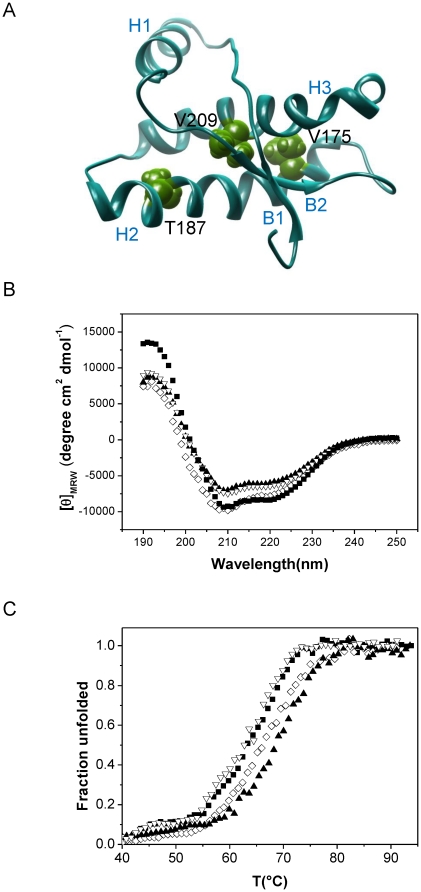
V175, T187 and V209 in murine prion protein can be substituted for isoleucine without large influence on protein secondary structure. A) Schematic representation of amino-acid residues selected for substitution with more hydrophobic isoleucine. V175 and T187 reside in H2 and V209 lies in H3. B) CD spectra of wild-type (▪), V175I (⋄), T187I (▴), and V209I (▿) reveal a high content of α-secondary structure as expected for mPrP. C) Thermal denaturation curves of wild-type and mutants (symbols used as above).

Several amino-acid substitutions increasing side chain hydrophobicity (e.g. V210I, T180A, T183A) lead to spontaneous prion disease in humans. In this study we focused on the subdomain H2-H3, which is tethered by the native disulfide bridge and where many human pathogenic mutations reside. In addition to the V209I mutant, which corresponds to the fCJD-causing mutation V210I in humans, we selected two residues within the same subdomain of PrP for mutagenesis. According to the determined mPrP structures [15], [16] side-chains of residues V175 and T187 residing in H2 are oriented toward H3 (Figure 1A). Amino-acid residues V175, T187 and V209 are conserved among different mammalian prion proteins. Substitution of residues V175 or T187 for a more hydrophobic isoleucine should increase the hydrophobic interactions between H2 and H3. There are no known pathogenic mutations of V176 (corresponding to V175 in mPrP), whereas very rare mutations of T188 to alanine, lysine or arginine (corresponding to T187 in mPrP) were linked to hereditary prion disease in humans [17]. In the mPrP structure V209 in H3 points towards the interface of B2 (beta strand 2) and H3 and takes part in the hydrophobic core, thus contributing to stabilization of the overall fold. Since the side chains of V175I and T187I are limited to the subdomain H2-H3 and do not interact with the other subdomain, we anticipated differences in the effect of different hydrophobic mutations on conversion.

*In vitro* studies of recombinant prion protein provided information on the structure and stability of PrP^C^ [1] and insights into the mechanism of PrP conversion [8], [11], [18]. Moreover, *in vitro* converted bacterially expressed prion protein was shown to be infectious to wild-type animals [19], [20], [21] conclusively proving relevance of the *in vitro* conversion system. We therefore compared biophysical properties and fibrillization propensities of recombinant mPrP harboring single point mutations V175I, T187I and V209I to wild-type mPrP. mPrPs carrying selected mutations were expressed in form of inclusion bodies in bacteria and successfully refolded. Characteristic minima at 208 nm and 222 nm in far-UV CD spectra demonstrated that wild-type protein and all three mutants had predominantly alpha-helical structure (Figure 1B) indicating that mutations did not disrupt the protein fold. We were further interested whether the introduced hydrophobic mutations influenced the thermal stability of the protein. V209I mutant did not have any influence on protein stability, as has been observed previously [4]. In contrast thermal stability of T187I and V175I was increased in comparison to both the wild-type and V209I mPrP (Figure 1C, Table 1).

**Table 1:** Thermal stability of mutant prion proteins.

Protein	T_m_ (°C)
Wild-type	64.7±0.8
V175I	67.2±.1
T187I	68.6±1.0
V209I	64.6±1.3

### Hydrophobic mutants display different conversion properties *in vitro*

**Figure 2:**
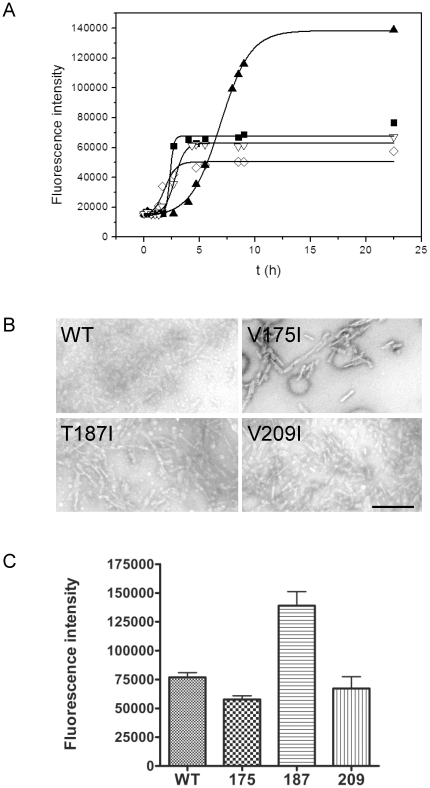
*In vitro* conversion of isoleucine mutants. A) *In vitro* fibrillization of wild-type (▪), V175I (⋄), T187I (▴), and V209I (▿) mPrPs followed by fluorescence intensity of thioflavin T. A representative of four experiments is shown. B) Presence of fibrils was confirmed by TEM. Bar represents 250 nm. C) Final thioflavin T fluorescence intensity of T187I is significantly higher than in the samples of other mPrPs (p<0.0005). The differences among wild-type, V175I and V209I are not significant.

*In vitro* fibrillization of hydrophobic mPrP mutants was monitored by an increase of fluorescence of an amyloid specific dye, thioflavin T (ThT) [22]. All prepared hydrophobic mutants converted into the amyloid form (Figure 2A). In the time course of conversion V175I slightly preceded fibril formation by wild-type mPrP and V209I whereas mPrP T187I displayed an increase in lag phase (Figure 2A). Negatively stained fibrils were present in samples of all mPrP variants as observed under the transmission electron microscope (Figure 2B). As in our previous study [11] we concluded that there is no simple correlation between the stability of PrP and lag phase in the fibrillization assay. V175I and T187I were both more stable than wild-type, yet they reproducibly fibrillized with either shorter or longer delay than the wild-type mPrP, respectively. Surprisingly, the T187I mutant, which had the longest fibrillization lag phase, exhibited significantly higher ThT fluorescence intensity than other mPrPs (P<0.0005) (Figure 2C). Although ThT is the most commonly used dye for detection of amyloids, its mechanism of binding has not been resolved [23]. The amount of proteinase K-resistant prion protein was not significantly different between wild-type and T187I samples (not shown). Higher ThT fluorescence intensity could thus originate from different types of amyloid structures formed as previously observed [24], [25].

### Presence of thin fibrils in T187I fibrillization reactions

**Figure 3:**
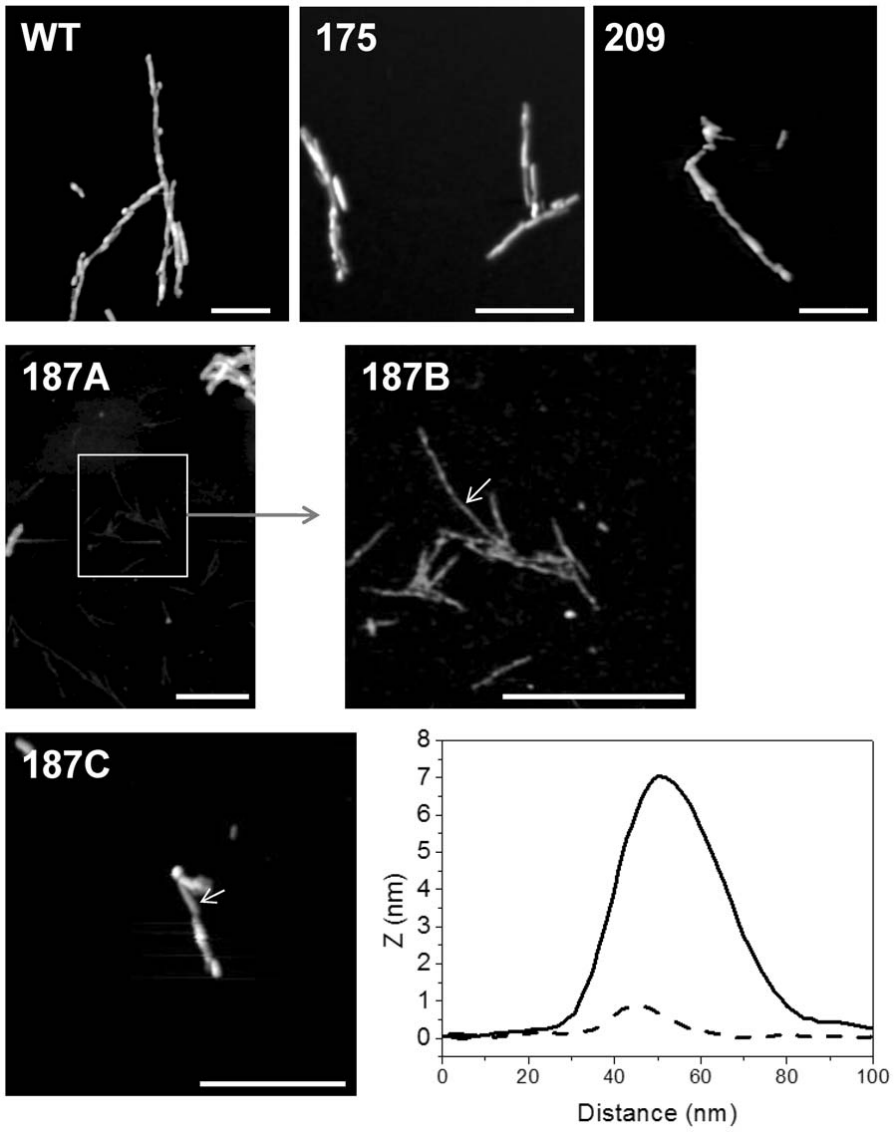
AFM reveals the presence of thin fibrils in addition to mature fibrils in conversion reactions of T187I. Conversion reactions of wild-type (WT), V175I, V209I (upper row) and T187I (middle, bottom row) were observed under the atomic force microscope. In all samples fibrils with approximate height around 7 nm were observed. Cross-section of such fibril from sample T187I is shown (bottom row right, full line). In the T187I sample in addition to such fibrils, fibrils with less than 1 nm in height were also present (middle row). Image 187B shows a close-up of selected part in image 187A. Cross-section of thin fibril is shown in the bottom row (bottom row right, broken line). Bar represents 500 nm. Arrows in images 187B and 187C indicate positions where cross-sections were taken. Images have not been corrected for the width and shape of the AFM tip.

We wanted to explore the cause of differences in conversion of PrP hydrophobic mutants. End-point fibrillization reactions (when ThT fluorescence intensity reached maximum) were therefore observed by atomic force microscopy. Diverse fibrillar structures (e.g. branched, twisted amyloid fibrils) were observed in the samples of wild-type, V175I and V209I mPrPs (Figure 3, upper row) and to smaller extent also in the sample of T187I (Figure 3, 187C). Fibrils in samples from wild-type mPrP, V209I and V175I were approximately 7 nm thick. In T187I sample we observed in addition to the thick fibrils also very thin fibrillar structures (Figure 3, 187 A,B). The height of these fibrillar structures was almost ten times lower than of thick fibrils (Figure 3, bottom row right). It is difficult to precisely determine the height of fibrils using AFM considering the effects of adsorption or sample drying, however clearly thinner fibrils in V187I sample probably correspond to the thinnest fibrillar structures identified previously [26], which were proposed to consist of a single filament. These fibrillar structures or filaments have not been observed at the end stage of fibrillization of wild-type, V175I and V209I mPrPs, suggesting that T187I substitution either hinders annealing of filaments into thick fibers or increases the stability of these thin fibrils, which may lead to their increased number.

### T187I forms increased amounts of PrP^res^ when exposed to 22L mouse prion strain in cell culture

**Figure 4:**
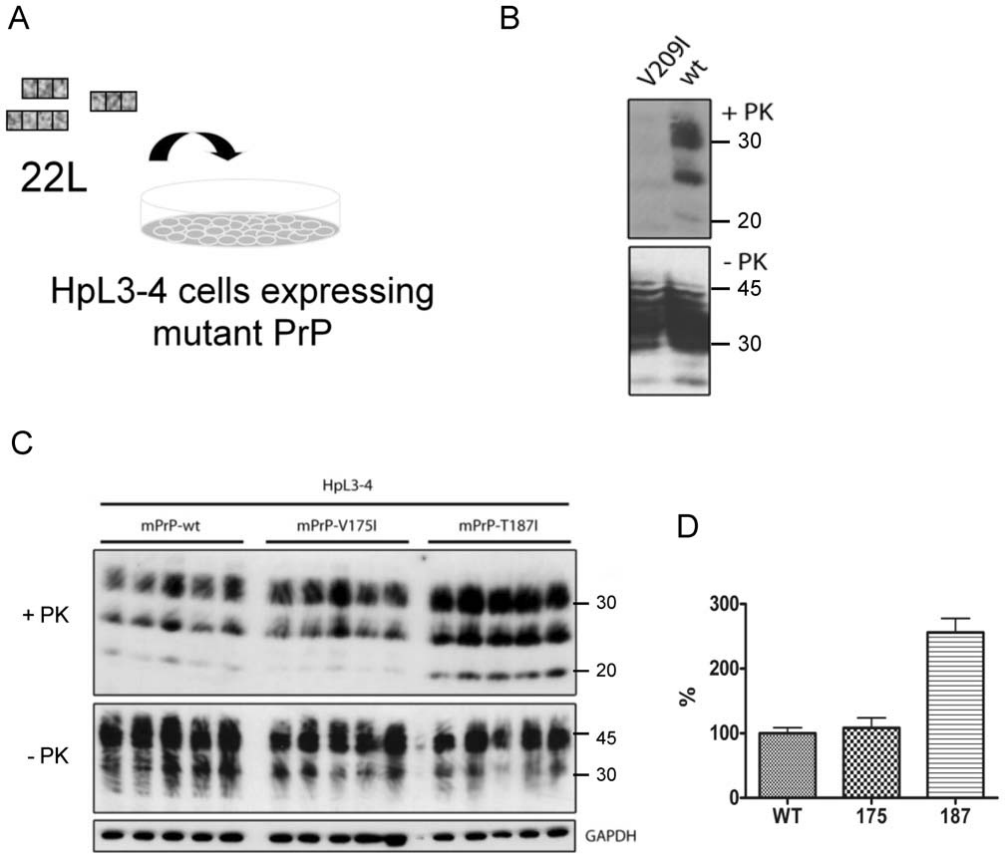
Cell line HpL3-4 expressing T187I produces more PrP^res^ than cell lines expressing wild-type and other mutants when infected by mouse prion strain 22L. A) Stably transduced HpL3–4 bulk populations expressing wild-type and mutant PrP proteins were exposed to mouse-adapted prion strain 22L and subsequently passaged in the absence of inoculum for 4 passages. B) Cell lysates of HpL3–4 cell populations expressing wild-type mPrP or mPrP V209I were tested for PrP^res^ content. While mutant V209I expressed well in HpL3–4 (non-digested by proteinase K, -PK), only weak bands of PrP^res^ are present in this cell population (+PK). C) Wild-type mPrP, mPrP V175I and mPrP T187I-expressing HpL3–4s were infected by 22L prions (5 parallels each). Upper part shows PrP^res^ (+PK), middle part non-digested cell lysate (-PK) and bottom line GAPDH loading control. D) Analysis of averaged normalized intensity of PrP^res^ reveals significantly higher amount of PrP^res^ present in cell lysates of HpL3–4s expressing T187I (p<0.0001).

To address the question if hydrophobic mutations in mouse PrP would also support PrP^res^ formation, wild-type mouse PrP and hydrophobic PrP mutants were also expressed in PrP deficient hippocampal cell line HpL3–4 [27]. Cells were stably transduced with pseudotyped retrovirus coding for wild-type and mutant mPrP and bulk populations of cells were tested for PrP^C^ expression. PrP mutants and wild-type mPrP were expressed at comparable levels on the cell surface as determined by flow cytometry (Table 2). Cell populations were then exposed to murine prion strain 22L (Figure 4A). After four passages cells were checked for the presence of proteinase K-resistant prion protein (PrP^res^), the marker of prion infection. Only traces of PrPres were detected in HpL3–4 cell line stably expressing V209I mutant, from which we concluded that this mutant poorly supports 22L prion propagation (Figure 4B). On the contrary, mPrP mutants V175I and V187I allowed efficient 22L prion propagation as detected by distinctive bands in Western blots after proteinase K digestion (Figure 4C). Furthermore, the T187I mutant reproducibly produced more than twice the amount of PrPres than wild-type mPrP or V175I (Figure 4D, p<0.0001) suggesting that T187I mutation either increased the susceptibility to 22L prion strain or imposed formation of more proteinase K-resistant aggregates upon 22L infection.

**Table 2:** Surface expression of mutant prion proteins in prion protein knockout cell line HpL3–4.

HpL3–4 mPrP	% PrP positive cells	X-mean
Wild-type	74	41
V175I	67	32
T187I	73	44
V209I	57	42

Flow cytometry analysis confirmed surface expression of mutant and wild-type mPrP. The number of PrP positive cells as well as the mean fluorescent intensity of positive cells is given.

## Discussion

The aim of this study was to investigate the role of hydrophobic mutations in the H2–H3 subdomain of the globular domain of PrP. Several hydrophobic mutations in the globular domain are associated with human prion diseases. Aberrant processing of several of those mutations (e.g. V180I) was observed in cell culture experiments [6]. Several mutants also showed decreased thermodynamic stability of PrP^C^ or increased stability of the folding intermediate when studied *in vitro* [5]. Previously we have shown that separation of subdomain B1-H1-B2 from subdomain H2–H3 is necessary for prion protein conversion while both subdomains retain the arrangement of their secondary structure elements [11]. Mutation V209I is associated with familial CJD (corresponding to human PrP amino acid residue 210) and this residue is tightly embedded in the hydrophobic core and might affect both subdomain separation as well as their reannealing. On the other hand, mutations in H2 with increased hydrophobicity, V175I and T187I, should stabilize subdomain H2–H3, and might thus facilitate separation of subdomains and conversion. Substitution of valine for isoleucine at position 209 in mPrP did not affect its stability, consistent with findings of a previous study [4]. The effect of this mutation was proposed to lead to increased stability of the folding intermediate [5]. Molecular dynamics simulations proposed that isoleucine might cause steric crowding in the hydrophobic core which may cause misfolding [28], however we observed indistinguishable *in vitro* conversion. Surprisingly, the mPrP V209I mutant was inefficiently converted to PrP^res^ in HpL3-4 cells exposed to mouse prion strain 22L. Direct prion titer analysis of different cell populations could not be performed due to expected transmission barriers introduced by PrP amino acid substitutions. However, the fact that PrP^res^ was present in all cell lines several passages post infection argues that all PrP mutants were capable of supporting prion infection, albeit likely to different degrees.

PrP mutation V210I (corresponding to V209I in mouse PrP) is associated with genetic prion disease in humans, arguing that this mutation *per se* is not refractory to prion formation. A likely explanation is that prion strains differ in their capacities to refold a given mutant PrP^C^ into its infectious isoform [29], [30]. Furthermore, differences in the human and murine PrP amino acid sequence context can influence the conversion efficiency of PrP^C^, as has been shown for other PrP mutants [31]. The fact that a pathogenic PrP amino acid substitution does not generally support efficient PrP^res^ formation once again demonstrates different degrees of compatibility of PrP mutants with different PrP^Sc^ conformers [30]. Previously identified mutations which did not support propagation of 22L prions were mapped to the surface and were proposed to interfere with packing of PrP^Sc^ aggregates [32], whereas V209I lies in the hydrophobic core. Previously observed differences in the population of the folding intermediate may be the reason that conversion of this mutant under the physiological conditions proceeds slowly or towards the nonfibrillar aggregates, while *in vitro* strongly unfolding conditions decrease this difference.

Two hydrophobic PrP mutants V175I and T187I displayed increased thermal stability in comparison to the wild-type protein, indicating that introduced isoleucines interact favorably with H3 and extend the hydrophobic core. Substitution of valine for isoleucine at position 175 of mPrP did not appreciably alter *in vitro* fibrillization kinetics. No overt differences in structure between wild-type and V175I PrP fibrils were observed by atomic force microscopy. Furthermore, PrP mutant V175I efficiently supported PrP^res^ formation upon 22L prion infection when expressed in HpL3-4 cells. These results are consistent with the hypothesis that stabilization of the subdomain H2-H3 by hydrophobic amino acid substitutions does not interfere with the PrP conversion process.

Interestingly, T187I exhibited an increased lag phase but a significantly higher amplitude of ThT response compared to wild-type PrP. We attribute this increased ThT response in T187I fibrillization at least in part to the formation of very thin fibrils. A morphological diversity has been previously observed in scrapie-associated fibrils extracted from prion-infected brains [33], [34] and *in vitro* prepared fibrils [26]. These thin fibrils of T187I mutant probably correspond to the previously identified single filament fibrils, which were very rare in the samples of wild-type mPrP [26]. In accordance with increased *in vitro* conversion, when cells expressing T187I were infected with 22L prions, significantly more PrP^res^ was produced than in cells expressing wild-type or V175I mutant. Cell lines expressing the T187I mPrP mutant might represent good candidates for development of a cell assay with increased sensitivity. As the increased hydrophobicity at position 187 increases the stability of the globular domain, enhanced conversion cannot be caused by increased hydrophobic interaction between PrP monomers. Thr187 is the most exposed to the solvent of the three investigated mutants. Therefore its replacement by a more bulky isoleucine residue may prevent efficient lateral packing of converted filaments into thick fibrils, and maintain a higher number of nuclei for conversion.

## Materials and Methods

### Materials

The anti-PrP mouse monoclonal antibody 4H11 has been described previously [35]. Cy2-conjugated anti-mouse antibodies were purchased from Dianova, goat anti-mouse HRP conjugated antibodies from Jackson ImmunoResearch, and Ni-NTA resin was from Qiagen. TEM grids were from SPI Supplies, mica was from Ted Pella, GdnHCl and urea were purchased from Fluka, cell culture media and other chemicals were form Gibco (Invitrogen). All other chemicals were purchased from Sigma.

### Preparation of PrP mutant constructs

For expression in *Escherichia coli,* single amino acid substitutions were introduced into the mPrP open reading frame (from residues 23 to 230) cloned into plasmid pRSET A using the Quikchange kit. Substitutions were also introduced into the mPrP open reading frame in pcDNA3.1 zeo (+) by site-directed mutagenesis and these open reading frames were further subcloned into the retroviral expression vector pSFF using the restriction enzyme sites BamHI and EcoRI [36], [37], [38].

### Protein expression, purification, and refolding

Plasmid pRSET A encoding mPrP mutants was transformed into competent *E. coli* BL21 (DE3) pLysS. Protein expression was induced by 1 mM IPTG. 4 h after induction cells were harvested by centrifugation. The protein was purified from inclusion bodies and refolded on a Ni-NTA-column using a previously described protocol [39], [40], [41]. The purity of mutant isolates was checked by SDS-PAGE.

### Circular dichroism spectroscopy

Circular dichroism spectra were recorded on an Applied Photophysics Chirascan spectropolarimeter. Far-UV CD spectra were recorded between 190 and 250 nm in a 1 mm path length cuvette at a protein concentration of 0.1 mg/ml. The thermal stability of proteins was recorded in a 1 mm path length cuvette at protein concentrations of 0.1 mg/ml with a temperature scan rate of 1°C/ min at 222 nm.

A s previously described [42] T_m_ was obtained from the thermal transition curves by a fit to the equation:
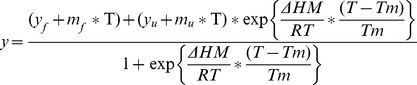
where y is the observed circular dichroism, (* y_f_* + m_f_*T ) and (* y_u_* + m_u_*T ) describe the linear dependence of the pretranslational and posttransitional baselines on temperature, ΔH_m_ is the enthalpy change for unfolding at T_m_, and T_m_ is the midpoint of the thermal unfolding curve. Two-state model equation described above was implemented into the program Origin 8.1 (MicroCal, GE Healthcare).

### *In vitro* conversion of the prion protein

A conversion reaction adopted from Bocharova et al. [43] was used for tracking the fibrillization of PrP mutants. Correctly folded proteins were first denatured in 6 M GdnHCl and further diluted into 1 M GdnHCl, 3 M urea, phosphate buffered saline pH 6.8 at protein concentrations of 22 µM. Amyloid specific dye Thioflavin T (5 µM) was added and the samples were loaded into microtiter wells each containing 3 teflon balls [43]. Microtiter plate was shaked at 1000 rpm at 37°C and thioflavin T fluorescence intensity (at 510 nm) was followed by Mithras LB 940 Multimode Microplate Reader (Berthold Technologies) equipped with 440 nm excitation filter. Statistical analysis was performed in Graphpad Prism using one-way nonparametric ANOVA test.

### Analysis of fibril formation

Transmission electron microscopy was carried out as previously described [40]. To observe conversion reaction by atomic force microscopy a drop of fibrillization mixture (diluted to 0.22 µM) was applied to freshly cleaved mica (Ted Pella) and left to adsorb for 5 min after which it was washed twice with filtered Milli-Q water and dried under nitrogen. Samples were observed by Agilent Technologies 5500 Scanning Probe Microscope operating in acoustic alternating current mode utilizing silicon cantilevers (Arrow-NCR, NanoWorld) as previously described [11].

### Production of retrovirions and transduction of HpL3-4

The pSFF vector and the packaging plasmids pVPack-GP and pVPack-VSV-G (Stratagene, La Jolla, CA, USA) were transfected into HEK293-FT (Invitrogen) cells using Lipofectamine 2000 according to the manufacturer's recommendations. Retroviral supernatants were harvested 48 h post transfection and cleared of cell debris by filtration through a 0.45 µm filter. Retroviral supernatants were diluted in growth medium and mixed with DEAE-Dextran or polybrene to a final concentration of 10 µg/ml or 4 µg/ml, respectively. HpL3-4 cells were incubated with retroviral particles for 3 h at 37°C. After addition of 2 ml growth medium cells were incubated for additional 24 h. Cells were then rinsed, expanded and subsequently tested for PrPC expression.

### Flow cytometry

We used a flow cytometry protocol adapted from Maas *et al.* to check whether PrP hydrophobic mutants are expressed at the cell surface of HpL3-4 cells similarly to the wild-type PrP [32]. 5×10^5^ cells were first incubated with FACS buffer (2.5 % FCS in PBS) for 10 min at 4°C. 100 µl of 4H11 antibody was added to the cells and incubated for 45 min at 4°C. After washing, the cells were incubated with Cy2-conjugated anti-mouse secondary antibodies for 45 min at 4°C in the dark. The rinsed cells were analyzed by flow cytometry.

### Infection of HpL3-4

A day before infection 5×10^4^ cells were seeded into each well of a 24-well microtiter plate. 200 µL of DMEM supplemented with 10% serum and 1% 22L-positive brain homogenate were added to the cells and incubated for 4 h. After this incubation 400 µL of growth medium were added and the cells were left to grow to near confluence when the cells were subsequently expanded into 6 cm and 9 cm Petri dishes [32], [44].

### Analysis of protease-resistant PrP

Infected HpL3-4 cells were rinsed in PBS and lysed in cold lysis buffer (10 mM Tris–HCl pH 7.5, 100 mM NaCl, 10 mM EDTA, 0.5% Triton X-100, 0.5% DOC). Cell lysates were cleared of cell debris (20 000x g, 1 min). 1/10 of the sample was precipitated in four volumes of methanol and used for determination of the total amount of PrP. The remaining sample was incubated with proteinase K (20 µg/ml) at 37°C for 60 min. Proteolysis was terminated by addition of Pefabloc and lysates were centrifuged at 356.000xg for 1 h at 4°C. Pellets were resuspended in SDS-sample buffer (2.5% SDS, 3 mM EDTA, 2% β-mercaptoethanol, 5% glycerol, 0.02% bromphenol blue and 63 mM Tris-HCl pH 6.8 and analyzed by Western blot. PrP was detected by the mouse monoclonal antibody 4H11 and enhanced chemiluminescence according to the manufacturer's recommendations (ECL Plus, Amersham Biosciences). Bands corresponding to PrP^res^ were analyzed by densitometry and normalized to control GAPDH. As in the case of *in vitro* conversion statistical analysis was performed with Graphpad Prism using one-way nonparametric ANOVA.
